# Modulations of wheat growth by selenium nanoparticles under salinity stress

**DOI:** 10.1186/s12870-024-04720-6

**Published:** 2024-01-08

**Authors:** Sara Zafar, Zuhair Hasnain, Subhan Danish, Martin Leonardo Battaglia, Shah Fahad, Mohammad Javed Ansari, Sulaiman Ali Alharbi

**Affiliations:** 1 Botany Department, Government College University, Faisalabad, Pakistan; 2 PMAS Arid Agriculture University, Rawalpindi, Pakistan; 3 Department of Soil Science, Faculty of Agricultural Sciences and Technology, Bahauddin Zakariya University, Multan, Punjab Pakistan; 4 The Nature Conservancy, Arlington, VA 22203 USA; 5 Department of Agronomy, Abdul Wali Khan University Mardan, Mardan, Khyber Pakhtunkhwa 23200 Pakistan; 6 Department of Natural Sciences, Lebanese American University, Byblos, Lebanon; 7 Department of Botany, Hindu College Moradabad (MJP Rohilkhand University Bareilly), Moradabad, 244001 India; 8 Department of Botany and Microbiology, College of Science, King Saud University, PO Box -2455, Riyadh, 11451 Saudi Arabia

**Keywords:** Selenium nanoparticles, Biological synthesis, Antioxidants, Growth, Wheat plants

## Abstract

Salinity stress is a prominent environmental factor that presents obstacles to the growth and development of plants. When the soil contains high salt concentrations, the roots face difficulties in absorbing water, resulting in water deficits within the plant tissues. Consequently, plants may experience inhibited growth, decreased development, and a decline in biomass accumulation. The use of nanoparticles has become a popular amendment in recent times for the alleviation of salinity stress. The study investigated the biological approach for the preparation of Se nanoparticles (NP) and their effect on the growth of wheat plants under saline conditions. The leaf extract of lemon (*Citrus limon* L.) was used for the green synthesis of selenium nanoparticles (Se-NPs). The synthesized NPs were characterized by X-ray diffraction (XRD) and Fourier-transform infrared spectroscopy (FTIR) and were applied foliar in the range of 0.01%, 0.05% and 0.1% on wheat plants. Results showed that 0.1% SeNP alone exhibited a significantly higher yield per plant, biomass per plant, 1000 grains weight, chlorophyll *a*, chlorophyll *b* and total chlorophyll over the SS (salt stress) control. A significant decline in MDA and H_2_O_2_ also validated the effectiveness of 0.1% SeNP over the SS control.

## Introduction

Salinity stress is a significant environmental factor that poses challenges to plant growth and development [1–3]. When plants are exposed to high salinity levels in their surrounding soil or irrigation water, it disrupts their normal physiological processes, leading to various detrimental effects [4, 5]. One of the primary impacts of salinity stress on plants is inhibited growth [6, 7]. High salt concentrations in the soil hinder water uptake by roots, leading to water deficits within the plant tissues [8]. This water deficit, coupled with osmotic stress caused by excess salts, reduces cell expansion and overall plant growth [9, 10]. As a result, plants may exhibit stunted growth, reduced shoot and root development, and a decrease in biomass accumulation [11–15]. Salinity stress also triggers oxidative stress in plants. The presence of high salt levels generates reactive oxygen species (ROS) within plant cells. ROS, including hydrogen peroxide (H_2_O_2_) and superoxide radicals (O2-), cause oxidative damage to cellular components such as lipids, proteins, and DNA. This oxidative stress disrupts normal cellular functions and can lead to cell death [16, 17]. High salt concentrations also interfere with chlorophyll synthesis and disrupt its stability. As a result, plants experiencing salinity stress often exhibit a decline in chlorophyll content, leading to reduced photosynthetic efficiency [2, 18].

To combat the detrimental effects of salinity stress, the use of nanoparticles is becoming center of attention [19]. Nanoparticles possess unique physicochemical properties that make them promising candidates for enhancing plant tolerance to salinity stress. One approach involves the application of nanoparticles for targeted delivery of essential nutrients and growth-promoting substances to plants [20]. Salinity stress often disrupts nutrient uptake and transport processes in plants, leading to nutrient deficiencies. By encapsulating nutrients within nanoparticles, their release can be controlled, ensuring efficient delivery to plant roots. This targeted nutrient delivery helps alleviate nutrient imbalances caused by salinity stress and promotes optimal plant growth and development [21]. Additionally, can act as carriers for beneficial compounds such as antioxidants and plant growth regulators. These nanoparticles can protect the encapsulated bioactive molecules from degradation and deliver them directly to the plant tissues [22].

Although many scientists have documented the effect of nanoparticles, the need to study the use of selenium nanoparticles (SeNPs) in the context of salinity stress arises from their specific application rate for cereals, especially wheat. By exploring the application of SeNPs, this study aims to contribute to the development of innovative approaches for enhancing wheat plant tolerance to salinity stress. The novelty of our work lies in the selection of SeNPs best application rate as a potential solution to mitigate the detrimental effects of salinity stress on plants. It is hypothesized that SeNPs will enhance plant growth, alleviate oxidative stress, improve chlorophyll content, and regulate ion balance in plants exposed to salinity stress.

## Materials and methods

The leaves of *Citrus limon* L. were thoroughly rinsed with distilled water on three separate occasions. After drying, 10 g of homogeneous leaves were finely chopped and immersed in 90 mL of distilled water. The mixture was then boiled at 90 °C for 20 min and subsequently filtered and centrifuged at 1500–2000 rpm for ten minutes. The resulting supernatant was collected and stored at 4 °C for later use in the synthesis of Se nanoparticles.

### Green synthesis approach of SeNPs

Selenium nanoparticles (SeNPs) were synthesized through a simple yet effective procedure. Initially, 20 mL of the filtered supernatant, obtained from the boiled and centrifuged *C. limon* L. leaves, was mixed with 80 mL of a 1 mM Na_2_SeO_4_ solution. This solution was then subjected to sonication at a temperature of approximately 60 °C for one hour [23].

### Nanoparticles characterization

The diameter of selenium nanoparticles (SeNPs) was calculated by using the X-ray diffraction.

### Seed germination

In this study, wheat seeds of the Ghazi variety were procured from the Ayub Agricultural Research Institute, Faisalabad. To ensure sterility, the seeds were treated with a 15% sodium hypochlorite solution for 15 min and then thoroughly washed with distilled water. Subsequently, ten sterilized seeds were sown in individual plastic pots, each containing 8 kg of soil with specific properties as described in Table 1. The sowing took place at the experimental station of Government College University Faisalabad, Pakistan. The objective was to impose salinity stress on the wheat plants, and this was achieved by introducing NaCl solution (1%) prepared in distilled water to the soil two weeks after sowing. For the assessment of electrical conductivity EC meter was used. The target level of salinity was set at EC = 12 dS/m, developed by taking into account salinity of control (EC = 0.50 dSm^− 1^).

After 45 days of growth, exogenous foliar application of SeNPs was carried out on the wheat plants. Different concentrations of SeNPs, 0%, 0.01%, 0.05%, and 0.1%, were applied to the 45-days-old plants. To assess the effects of the application of SeNPs on the wheat plants, three plants were harvested from each plot 15 days after the application of nanoparticles. Various parameters were examined, including plant growth, antioxidant levels, and malondialdehyde (MDA) content. The harvested plants were subjected to oven-drying for 72 h to determine their dry weight.

**Table 1:** Physico-chemical properties of soil

Soil texture	Sandy-clay-loam	CO_3_^2−^ (meq/L)	Nil
ECe (dS/m)	0.60	HCO_3_^−^ (meq/L)	2.45
pH	7.6	Zn (µg/g)	1.90
Organic matter (%)	0.49	Available P (µg/g)	7.3
Saturation	36	Available K (µg/g)	40

The following Arnon [24] method was used to calculate chlorophyll (Chl) contents. The chlorophyll and carotenoid contents in freshly chopped leaves (0.5 g) were extracted using 10 mL of acetone (80%) at 20˚C overnight. The mixture was then centrifuged for 5 min at 14,000 g, and the optical density of the resulting supernatant was measured at three different wavelengths − 480 nm, 645 nm, and 663 nm - using a spectrophotometer (U-2800, Hitachi, Japan).

$$Chla = {\text{ }}\left[ {12.7{\text{ }}\left( {12.7{\text{ }}O{D_{663}}} \right){\text{ }}--{\text{ }}2.69{\text{ }}\left( {O{D_{645}}} \right)} \right]{\text{ }} \times {\text{ }}V/1000{\text{ }} \times {\text{ }}W$$

$$Chlb = {\text{ }}\left[ {22.9{\text{ }}\left( {O{D_{645}}} \right){\text{ }}--{\text{ }}4.68{\text{ }}\left( {O{D_{663}}} \right)} \right]{\text{ }} \times {\text{ }}V/1000{\text{ }} \times {\text{ }}W$$

$$Chlt{\text{ }} = {\text{ }}\left[ {20.2{\text{ }}\left( {O{D_{645}}} \right){\text{ }} + {\text{ }}8.02{\text{ }}\left( {O{D_{663}}} \right)} \right]{\text{ }} \times {\text{ }}V/1000{\text{ }} \times {\text{ }}W$$

$$Carotenoid{\text{ }}\left( {g{\text{ }}m{L^{ - 1}}} \right){\text{ }} = {\text{ }}Acar{\text{ }}/{\text{ }}Em{\text{ }} \times {\text{ }}100$$

Where Acar = OD_480_ + 0.114 (OD_663_) – 0.638 (OD_645_), Em x 100 = 2500, OD = optical density, V = sample extract volume and W = sample weight.

### Analysis of activity of antioxidant enzymes

In order to calculate the contents of antioxidant enzymes, 0.5 g leaves were homogenized in 50 mM phosphate buffer pH 7.8 and centrifuged at 15,000×g for 20 min at 4 ˚C. Supernatant was carefully shifted to a new tube and used for the determination of enzyme activity.

### Superoxide dismutase (SOD) activity

SOD activity was analyzed by following the method of Giannopolitis and Ries [25], based on inhibition of photochemical reduction of nitrobluetetrazolium (NBT) at λ = 560 nm. The reaction starts by adding 50 µL of the enzymatic extract to a mixture containing 50 µM NBT, 1.3 µM methionine, and 50 mM phosphate buffer at pH 7.8. The reaction mixture was then placed in a chamber under a 30 W fluorescent light source. The SOD activity was determined based on its ability to inhibit the reduction of NBT catalyzed by xanthine oxidase. As the SOD enzyme converts the superoxide radicals to hydrogen peroxide and molecular oxygen, it inhibits the reduction of NBT. The absorbance of the reaction mixture was measured using a UV-visible spectrophotometer at 560 nm. The definition of one SOD unit in this experiment is the amount of enzyme required to cause a 50% inhibition rate of NBT reduction.

### Catalase (CAT) activity

The reaction starts by adding 50 mM phosphate buffer pH 7, 5.9 mM H_2_O_2_, and 0.1 ml enzyme extract. After every 20 s, the absorbance was recorded at 240 nm. Each unit activity is change in the absorbance of 0.01 unit min^− 1^ [26].

### Peroxidase (POD) activity

In this study, the peroxidase activity of wheat plants treated with selenium nanoparticles (SeNPs) was evaluated by monitoring the peroxidation of guaiacol and H_2_O_2_. The reaction mixture used for this assay consisted of 50 mM phosphate buffer pH 5, 20 mM guaiacol (100 mL), 40 mM H_2_O_2_ (100 mL), and 0.1 mL of the enzyme extract from the wheat plants. The experiment involved measuring the absorbance at 470 nm at regular intervals of every 20 s [26].

### Malondialdehyde (MDA) contents

To measure the MDA content, 0.5 g of chopped leaf tissue was extracted using 0.1% trichloroacetic acid (TCA) and then centrifuged at 12,000 × g for 10 min. Next, 1 mL of the resulting supernatant was combined with 4 mL of thiobarbituric acid (TBA) solution, prepared at a concentration of 0.5% in 20% TCA. The mixture was then heated at 95 ˚C in a water bath for 30 min. After the incubation, the sample was centrifuged for an additional 10 min. The absorbance of the samples was measured using a spectrophotometer at two specific wavelengths: 532 and 600 nm [27].

$$MDA level \left(nmol\right)=\varDelta ({A}_{532}nm-{A}_{660}nm)/1.56\times 105$$

MDA values were determined using an extinction coefficient of 156 mmol^− 1^ cm ^− 1^.

### Total free amino acids (TFA)

About 0.5 g of plant material was chopped in a 0.2 M buffer with pH 7.0. In a test tube, 1 mL of extract was added with 1 mL of ninhydrin (2%) and 1 mL of pyridine (10%). After heating for 30 min, the reaction mixture was cooled at normal temperature. Absorbance was determined at 570 nm using a spectrophotometer.

### Hydrogen peroxide (H_2_O_2_)

About 0.5 g of fresh leaves were chopped with 5 mL of TCA (0.1% w/w), and centrifuged for 15 min at 12,000 rpm. One mL of potassium iodide and 0.5 mL of phosphate buffer (pH 7.0) were added to about 0.5 mL of supernatant. Following vortexing, the absorbance at 390 nm was calculated using a UV-visible spectrophotometer [28].

### Yield attributes

Plant height, thousand-grain weight, plant biomass, and plant yield were calculated at the maturity of the crop.

### Statistical analysis

Data analysis was performed statistically using OriginPro 2021 [29]. It was also used for making paired comparison graphs with probability values. The least significant difference (LSD) test with a 5% probability level was used to observe the difference in means. Cluster plots with convex hull and hierarchical cluster plots were also made using OriginPro 2021.

## Results

### Characterization

**Fig. 1:**
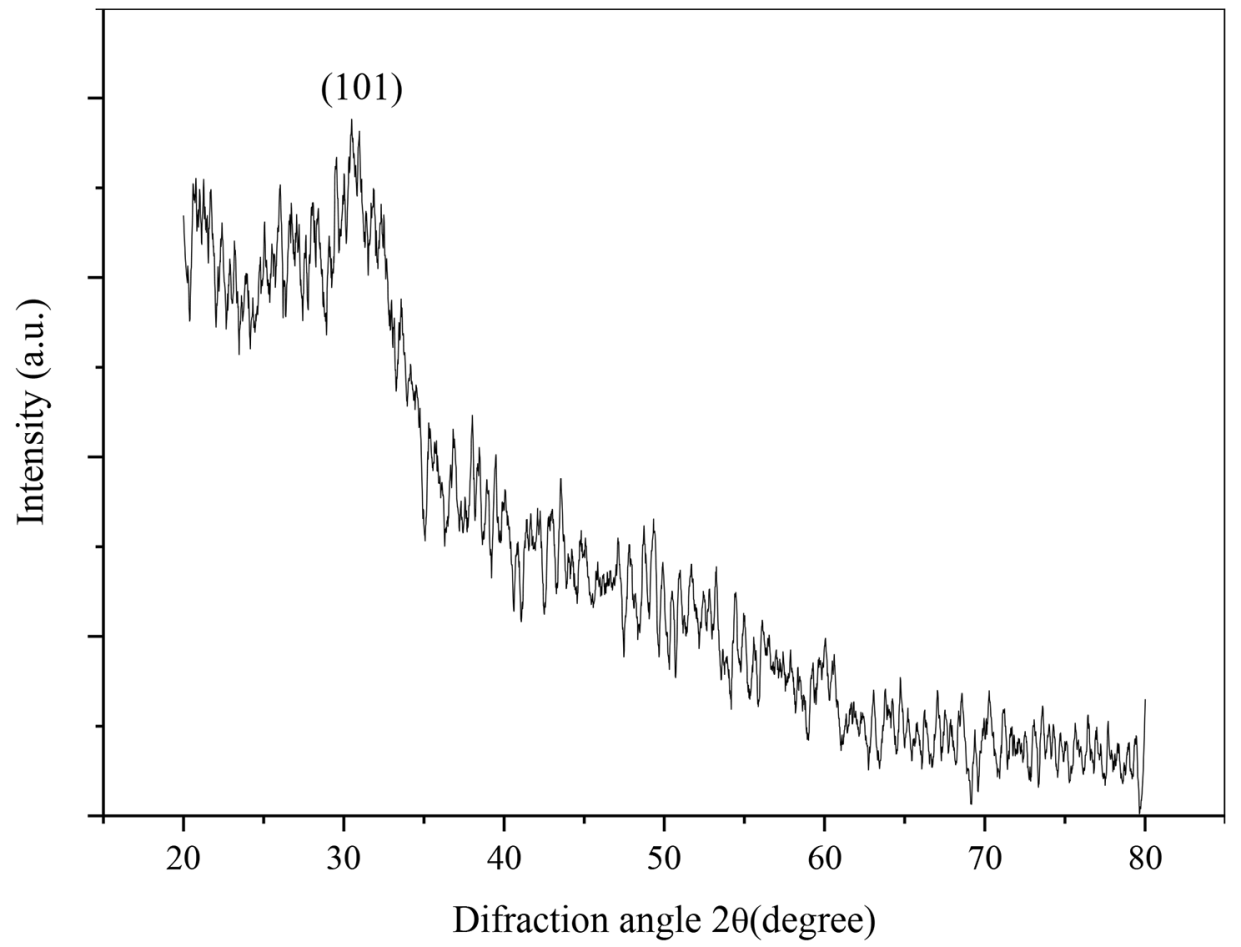
XRD pattern of SeNPs

The XRD scan covered a diffraction angle range from 20 to 80 degrees (Fig. 1). The resulting XRD plot provided conclusive evidence that the material exhibited a crystalline structure. Notably, a distinct and intense peak was observed at a diffraction angle of 30.62˚, corresponding to the (101) plane orientation. To determine the crystallite size of the prepared SeNPs, Scherrer’s formula (D = 0.9λ/βcosθ) was employed. In this formula, D represents the crystallite size, λ denotes the wavelength of the X-ray used (λ = 1.5406 Å), θ (in radians) represents the Bragg angle, and β stands for the full width at half maximum (FWHM, in radians) of the diffracted peak. Based on the calculations, the crystallite size of the synthesized SeNPs was found to be approximately 37 nm [23].

**Fig. 2:**
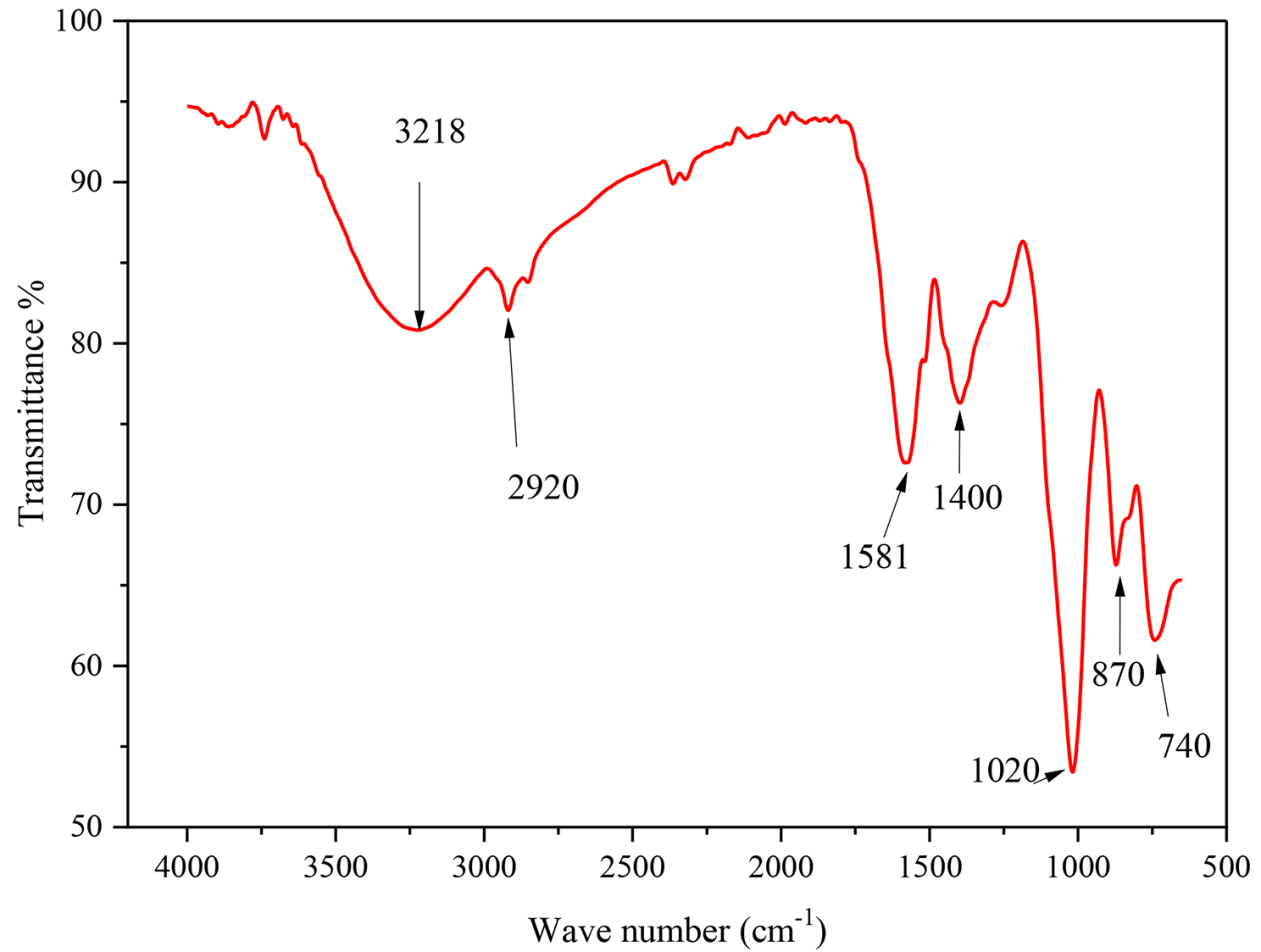
FTIR spectrum of SeNPs

By utilizing FTIR, researchers can identify the possible functional groups residing on the SeNPs’ surface, which play a crucial role in the reduction of selenate during the biochemically synthesized SeNPs formation process. In this study, a small amount of the prepared SeNPs was used as the sample specimen for FTIR analysis. The FTIR spectrum was collected in the range of wavelengths from 650 to 4000 cm^− 1^, with a resolution of 1 cm^− 1^. The obtained FTIR results revealed the presence of seven distinct peaks, each representing specific stretching and vibrational functions. The observed FTIR peaks occurred at wavelengths of 733.54, 867.19, 1018.42, 1394, 1584.68, 2914.82, and 3235.60 cm^− 1^ (Fig. 2). These peaks correspond to the following functional groups: aromatic C-H bending, C-H bending, C-N stretch, C-C stretch, N-O asymmetric stretch, free hydroxyl C-H stretch, and O-H stretch. The identification of these functional groups provides crucial insights into the surface chemistry and the roles of various compounds acting as capping agents on the SeNPs [23].

### Plant height

**Fig. 3:**
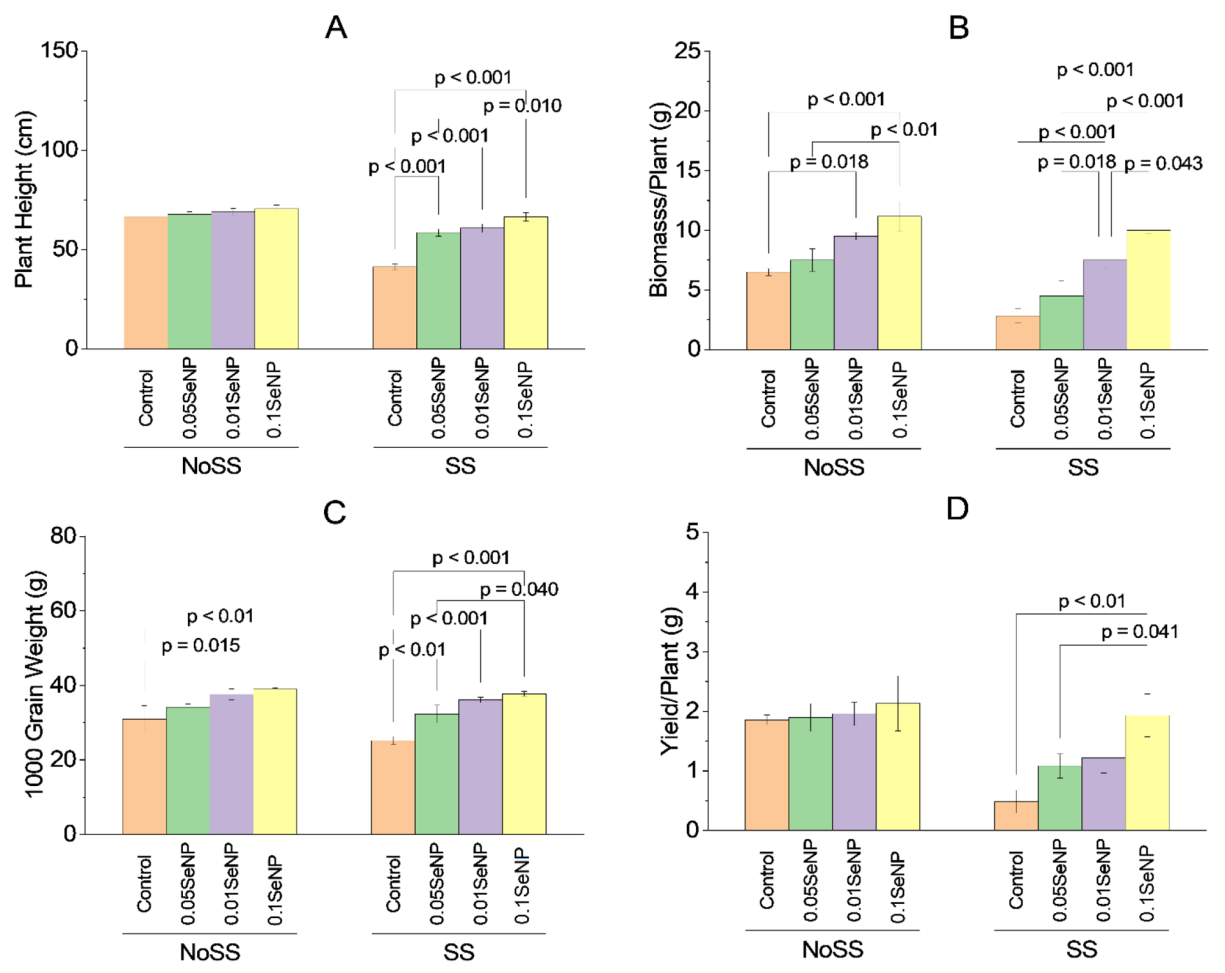
Effect of variable application rates of Se nanoparticles on plant height **(A)**, biomass/plant **(B)**, 1000 grains weight **(C)** and yield/plant **(D)**. Bars are means of 3 replicates. Different values on bars are probability values showing significant alteration at ≤ 0.05. SS = Salinity stress; NoSS = No salinity stress; SeNP = Selenium nanoparticles

In the no salinity stress (NoSS) condition, there were no significant differences in plant height between the control and 0.05% SeNP, 0.01% SeNP, or the combination of 0.01% SeNP and 0.05% SeNP (no-significant variation). Similarly, in the salinity stress (SS) condition, there were no significant variations in plant height between the control and 0.01% SeNP or the combination of 0.01% SeNP and 0.05% SeNP (no-significant variation). However, noteworthy findings emerged in the SS condition. In the SS control, the application of 0.05% SeNP resulted in a significant improvement in plant height over the control, with a *p*-value less than 0.001. Similarly, in the SS condition, the application of 0.01% SeNP led to a significant increase in plant height over the control, also with a *p*-value less than 0.001. Interestingly, when comparing the effects of different SeNP concentrations in the SS condition, no significant variations in plant height between 0.01% SeNP and 0.05% SeNP (no-significant variation) were found. However, in the SS condition, the application of 0.1% SeNP and 0.05% SeNP resulted in a significant improvement in plant height over the SS control, with a *p*-value 0.010. Overall, these results suggest that the application of SeNPs, particularly at concentrations of 0.05% SeNP and 0.01% SeNP, can positively influence plant height under salinity stress conditions. However, 0.1% SeNP and 0.05% SeNP showed the most pronounced effect in enhancing plant height in the presence of salinity stress (Fig. 3A).

### Biomass/plant

Under the NoSS condition, no significant variations in biomass/plant were noted between the NoSS control, 0.05% SeNP, 0.01% SeNP, and 0.1% SeNP. However, the NoSS experimental group treated with 0.01% SeNP displayed a statistically significant increase in biomass/plant growth over the NoSS control, with *p*-value 0.018. Similarly, in the SS.

condition, no significant variations were found in biomass/plant growth between the SS control and the SS experimental groups treated with 0.05% SeNP or the combination of 0.01% SeNP and 0.05% SeNP (no-significant variation). In contrast, the SS experimental groups treated with 0.1% SeNP exhibited significantly higher biomass/plant growth than the SS control group. The *p*-value was less than 0.001 for the SS experimental group treated with 0.1% SeNP alone and less than 0.01 for the SS experimental group treated with the combination of 0.1% SeNP and 0.05% SeNP. Regarding the effects of SeNP concentrations within the SS condition, no significant variations in biomass/plant growth were noted between the SS experimental groups treated with 0.05% SeNP and the control (no-significant variation). In contrast, the SS experimental group treated with 0.01% SeNP showed significantly higher biomass/plant growth over the SS control, with *p*-value 0.018. Moreover, the combination of 0.1% SeNP and 0.05% SeNP led to significantly increased biomass/plant growth over the SS control, with a *p*-value less than 0.001. Similarly, the SS experimental group treated with the combination of 0.1% SeNP and 0.01% SeNP demonstrated significantly higher biomass/plant growth over the SS control, with *p*-value 0.043. These findings indicate that the application of SeNPs, particularly at concentrations of 0.01% SeNP and 0.1% SeNP, can positively influence biomass/plant growth under both NoSS and SS conditions. Moreover, the combination of 0.1% SeNP and 0.05% SeNP exhibited notable effects in promoting biomass/plant growth in the presence of salinity stress (Fig. 3B).

### 1000 grains weight

In the context of the NoSS condition, no significant variations were noted in the 1000 grains weight between the NoSS control and 0.05% SeNP or the combination of 0.01% SeNP and 0.05% SeNP (*p* > 0.05). However, the NoSS experimental group treated with 0.01% SeNP exhibited a statistically significant increase in 1000 grains weight over the NoSS control (*p* = 0.015). Likewise, in the SS condition, the SS control displayed a significantly lower 1000 grain weight over the experimental group treated with 0.05% SeNP (*p* < 0.01). Additionally, the SS experimental group treated with 0.01% SeNP demonstrated a significantly higher 1000 grains weight over the SS control (*p* < 0.001). However, no significant variations were noted in the 1000 grains weight between the SS control and the experimental group treated with the combination of 0.01% SeNP and 0.05% SeNP (*p* > 0.05). In terms of the effects of SeNP concentrations within the SS condition, no significant variations were found in the 1000 grains weight between 0.05% SeNP or the combination of 0.01% SeNP and 0.05% SeNP, and the SS control (*p* > 0.05). However, the SS experimental groups treated with 0.1% SeNP alone or in combination with 0.01% SeNP displayed significantly higher 1000 grain weights than the SS control group. The *p*-value was less than 0.001 for the SS experimental group treated with 0.1% SeNP alone and 0.040 for the SS experimental group treated with the combination of 0.1% SeNP and 0.05% SeNP. These findings indicate that the application of SeNPs, particularly at a concentration of 0.01% SeNP, has a positive influence on the 1000 grains weight of plants under both NoSS and SS conditions. Moreover, the effects of 0.05% SeNP and 0.1% SeNP vary depending on the presence or absence of salinity stress (Fig. 3C).

### Yield/plant

No significant variations in the yield per plant were noted between the NoSS control and 0.05% SeNP, 0.01% SeNP, or the combination of 0.01% SeNP and 0.05% SeNP under the NoSS condition (*p* > 0.05). Similarly, in the SS condition, there were no significant variations in the yield per plant between the control and the SS experimental groups treated with 0.05% SeNP, 0.01% SeNP, or the combination of 0.01% SeNP and 0.05% SeNP (*p* > 0.05). However, within the SS condition, the SS experimental group treated with 0.1% SeNP alone exhibited a significantly higher yield per plant over the SS control (*p* < 0.01). Moreover, the SS experimental group treated with the combination of 0.1% SeNP and 0.05% SeNP demonstrated a statistically significant variation in yield per plant over the SS control (*p* = 0.041). These findings indicate that the application of SeNPs, particularly in conjunction with 0.1% SeNP, can positively influence the yield per plant under salinity stress conditions. However, there was no significant effect on the yield per plant when SeNPs were applied under the NoSS condition (Fig. 3D).

### Chlorophyll a

**Fig. 4:**
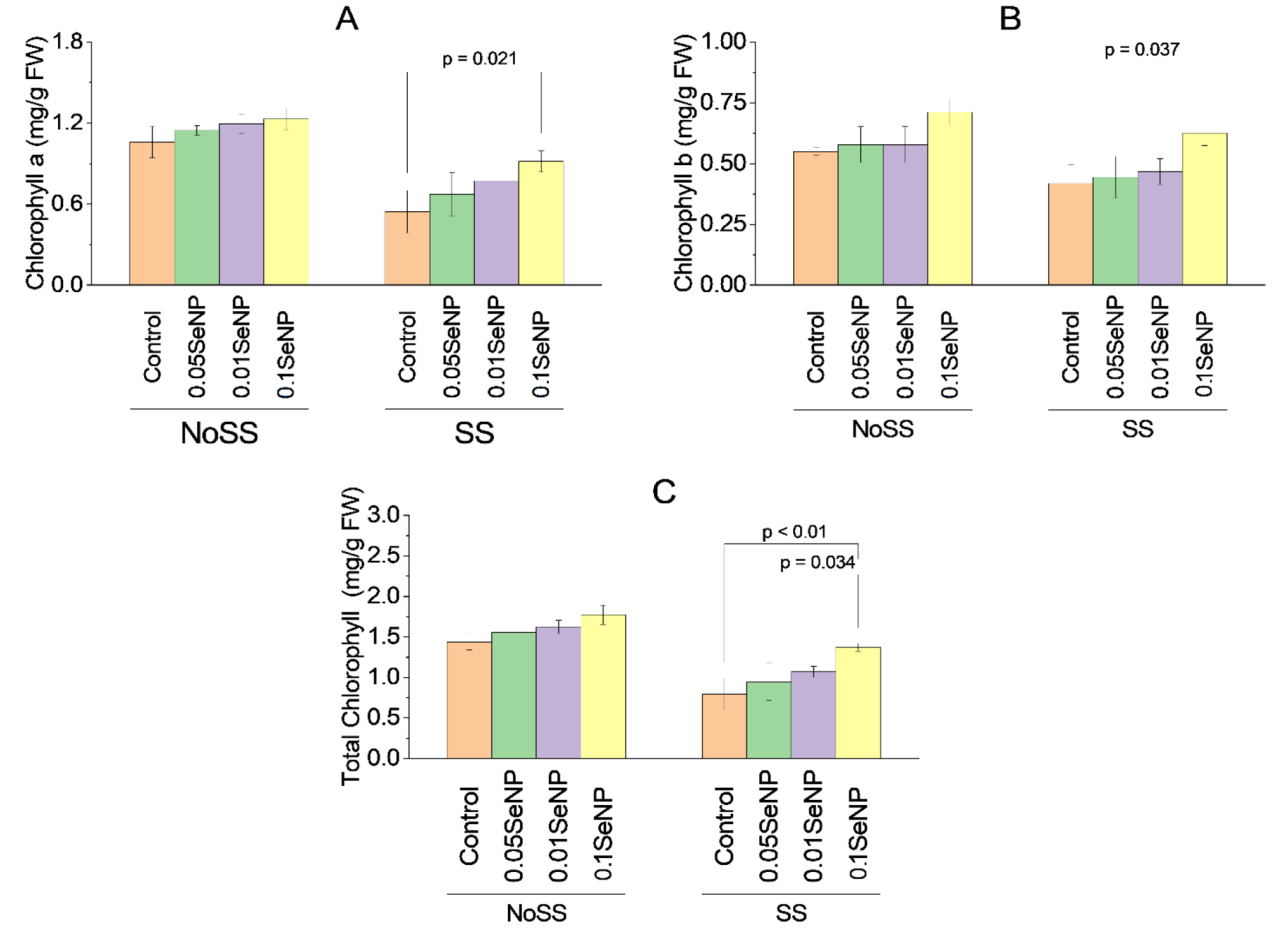
Effect of variable application rates of Se nanoparticles on chlorophyll a **(A)**, chlorophyll b **(B)** and total chlorophyll **(C)**. Bars are means of 3 replicates. Different values on bars are probability values showing significant alteration at ≤ 0.05. SS = Salinity stress; NoSS = No salinity stress; SeNP = Selenium nanoparticles

In the SS condition, there were no significant variations in chlorophyll a content between the SS control and the SS experimental groups treated with 0.05% SeNP, 0.01% SeNP, or the combination of 0.01% SeNP and 0.05% SeNP (*p* > 0.05). However, within the SS condition, the SS experimental group treated with 0.1% SeNP alone displayed a significant variation in chlorophyll a content over the SS control (*p* = 0.021). For the remaining experimental groups within the SS condition, no significant variations in chlorophyll a content were noted (*p* > 0.05). These findings suggest that the application of SeNPs did not have a significant impact on chlorophyll a content under both NoSS and SS conditions, except for the SS experimental group treated with 0.1% SeNP alone, which exhibited a significant variation (Fig. 4A).

### Chlorophyll b

The analysis of chlorophyll b content revealed no significant variations between the NoSS control and the NoSS experimental groups treated with 0.05% SeNP, 0.01% SeNP, or the combination of 0.01% SeNP and 0.05% SeNP under the NoSS condition (*p* > 0.05). Likewise, there were no significant variations in chlorophyll b content between the SS control and the SS experimental groups treated with 0.05% SeNP, 0.01% SeNP, or the combination of 0.01% SeNP and 0.05% SeNP under the SS condition (*p* > 0.05). However, in the SS condition, the SS experimental group treated with 0.1% SeNP alone exhibited a statistically significant variation in chlorophyll b content over the SS control (*p* = 0.037). Conversely, no significant variations in chlorophyll b content were noted among the remaining SS experimental groups (*p* > 0.05). These findings indicate that the application of SeNPs did not significantly affect chlorophyll b content under both NoSS and SS conditions, except the SS experimental group treated with 0.1% SeNP alone, which displayed a significant variation (Fig. 4B).

### Total chlorophyll

For total chlorophyll content, no significant variations were noted between the NoSS control and the NoSS experimental groups treated with various concentrations of selenium nanoparticles (SeNPs) under the NoSS condition (*p* > 0.05). Similarly, there were no significant variations in total chlorophyll content noted between the SS control and the SS experimental groups subjected to different SeNP treatments (*p* > 0.05). Nevertheless, within the SS condition, the SS experimental group treated with 0.1% SeNP alone exhibited a substantial increase in total chlorophyll content over the SS control, with a *p*-value lower than 0.01. Furthermore, the SS experimental group treated with the combination of 0.1% SeNP and 0.05% SeNP demonstrated a statistically significant variation in total chlorophyll content over the SS control, with *p*-value 0.034. These findings indicate that the application of SeNPs did not exert a considerable influence on total chlorophyll content under both NoSS and SS conditions, except for the SS experimental group treated with 0.1% SeNP alone and the combination of 0.1% SeNP and 0.05% SeNP, which exhibited significant variations (Fig. 4C).

### Catalase

**Fig. 5:**
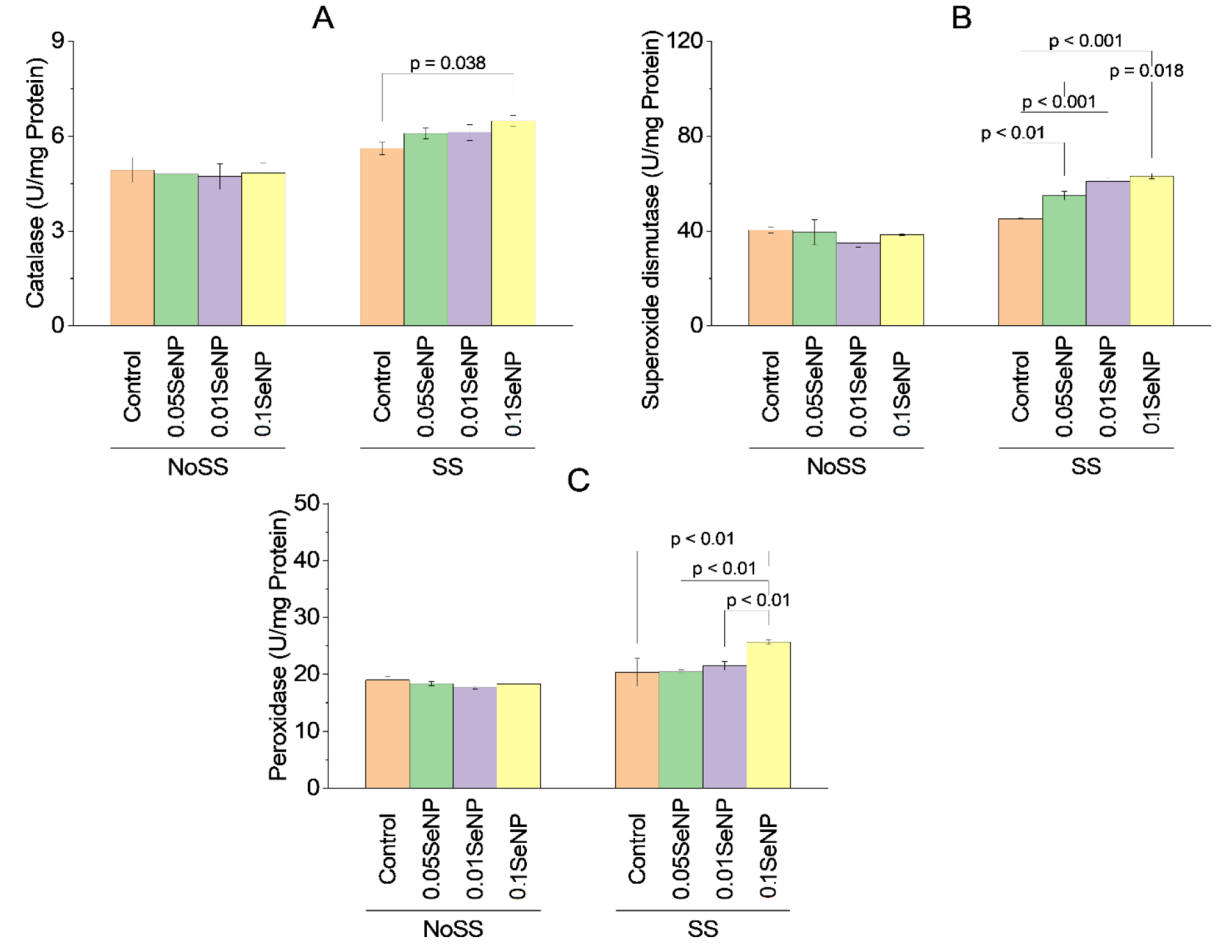
Effect of variable application rates of Se nanoparticles on catalase **(A)**, superoxide dismutase **(B)** and peroxidase **(C)**. Bars are means of 3 replicates. Different values on bars are probability values showing significant alteration at ≤ 0.05. SS = Salinity stress; NoSS = No salinity stress; SeNP = Selenium nanoparticles

There were no significant variations noted in catalase activity between the NoSS control and the NoSS experimental groups treated with 0.05% SeNP, 0.01% SeNP, or the combination of 0.01% SeNP and 0.05% SeNP under the NoSS condition (*p* > 0.05). Similarly, no significant variations in catalase activity were found between the SS control and the SS experimental groups treated with 0.05% SeNP, 0.01% SeNP, or the combination of 0.01% SeNP and 0.05% SeNP (*p* > 0.05). However, within the SS condition, the SS experimental group treated with 0.1% SeNP alone exhibited a statistically significant variation in catalase activity over the SS control, with *p*-value 0.038. For the remaining experimental groups within the SS condition, no significant variations in catalase activity were noted (*p* > 0.05). These findings suggest that the application of SeNPs did not significantly affect catalase activity under both NoSS and SS conditions, except for the SS experimental group treated with 0.1% SeNP alone, which exhibited a significant variation (Fig. 5A).

### Superoxide dismutase

There were no significant variations in superoxide dismutase (SOD) activity between the NoSS control and the NoSS experimental groups treated with 0.05% SeNP, 0.01% SeNP, or the combination of 0.01% SeNP and 0.05% SeNP under the NoSS condition (*p* > 0.05). Similarly, SOD activity did not significantly differ between the SS control and the SS experimental groups treated with 0.05% SeNP, 0.01% SeNP, or the combination of 0.01% SeNP and 0.05% SeNP under the SS condition (*p* > 0.05). However, in the SS condition, significant variations in SOD activity were noted. The SS experimental group treated with 0.05% SeNP showed significantly higher SOD activity over the SS control (*p* < 0.01). Likewise, the SS experimental group treated with 0.01% SeNP exhibited significantly higher SOD activity over the SS control (*p* < 0.001). No significant difference in SOD activity was noted between the SS experimental group treated with the combination of 0.01% SeNP and 0.05% SeNP and the SS control (*p* > 0.05). Furthermore, the SS experimental group treated with 0.1% SeNP alone demonstrated significantly higher SOD activity over the SS control (*p* < 0.001). Similarly, the SS experimental group treated with the combination of 0.1% SeNP and 0.05% SeNP showed a statistically significant variation in SOD activity over the SS control (*p* = 0.018) (Fig. 5B).

### Peroxidase

There were no significant changes in peroxidase activity relating the NoSS control and the NoSS experimental groups treated with 0.05% SeNP, 0.01% SeNP, or the combination of 0.01% SeNP and 0.05% SeNP under the NoSS condition (*p* > 0.05). Similarly, no significant variations in peroxidase activity were noted between the SS control and the SS experimental groups treated with 0.05% SeNP, 0.01% SeNP, or the combination of 0.01% SeNP and 0.05% SeNP under the SS condition (*p* > 0.05). However, within the SS condition, significant variations in peroxidase activity were noted. The SS experimental group treated with 0.1% SeNP alone exhibited significantly higher peroxidase activity over the SS control (*p* < 0.01). Similarly, the SS experimental group treated with the combination of 0.1% SeNP and 0.05% SeNP showed significantly higher peroxidase activity over the SS control (*p* < 0.01). Additionally, the SS experimental group treated with 0.1% SeNP and 0.01% SeNP also displayed significantly higher peroxidase activity over the SS control (*p* < 0.01) (Fig. 5C).

### Total free amino acids

**Fig. 6:**
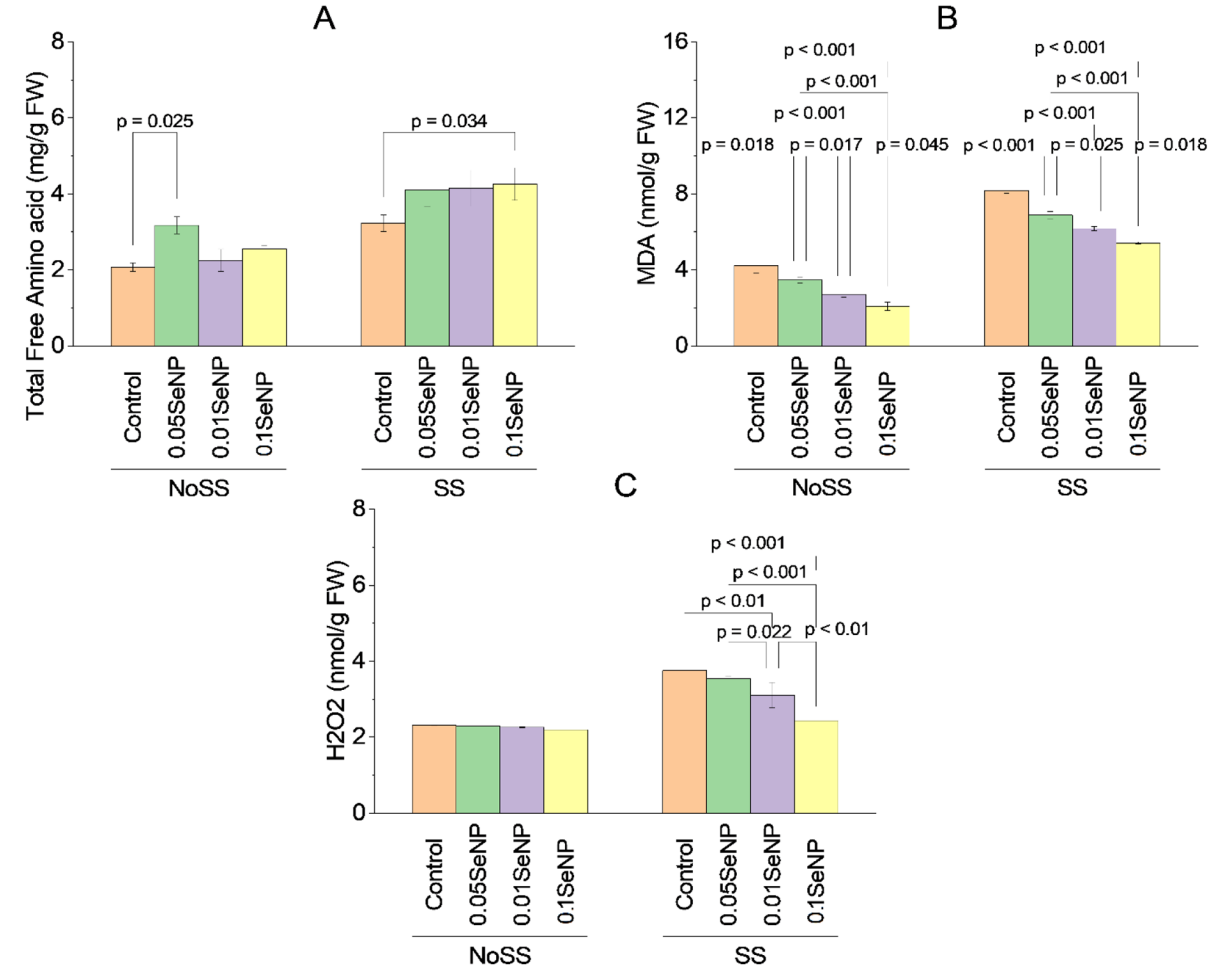
Effect of variable application rates of Se nanoparticles on total free amino acids **(A)**, MDA **(B)**, and H2O2 **(C)**. Bars are means of 3 replicates. Different values on bars are probability values showing significant alteration at ≤ 0.05. SS = Salinity stress; NoSS = No salinity stress; SeNP = Selenium nanoparticles

NoSS experimental group treated with 0.05% SeNP exhibited a significant variation in total free amino acid content over the NoSS control, with *p*-value 0.025. Similarly, no significant variations in total free amino acid content were found between the SS control and the SS experimental groups treated with 0.05% SeNP, 0.01% SeNP, or the combination of 0.01% SeNP and 0.05% SeNP under the SS condition (*p* > 0.05). However, the SS experimental group treated with 0.1% SeNP alone displayed a significant variation in total free amino acid content over the SS control, with *p*-value 0.034. These results indicate that the application of 0.05% SeNP in the NoSS condition and 0.1% SeNP in the SS condition had a significant impact on the total free amino acid content (Fig. 6A).

### MDA

For the NoSS condition, the NoSS experimental group treated with 0.05% SeNP exhibited a significant variation in MDA content over the NoSS control, with *p*-value 0.018. Similarly, the NoSS experimental group treated with 0.01% SeNP showed a significant variation in MDA content over the NoSS control, with *p*-value less than 0.001. Additionally, the NoSS experimental group treated with the combination of 0.01% SeNP and 0.05% SeNP displayed a significant variation in MDA content, with *p*-value 0.017. Similarly, the NoSS experimental group treated with 0.1% SeNP alone and 0.1% SeNP in combination with 0.05% SeNP both showed significant variations in MDA content over the NoSS control, with *p*-values of less than 0.001 and less than 0.001, respectively. However, no significant variation in MDA content was noted between the NoSS control and the NoSS experimental group treated with 0.1% SeNP and 0.01% SeNP (*p* = 0.045). Under the SS condition, the SS experimental group treated with 0.05% SeNP, 0.01% SeNP, and the combination of 0.01% SeNP and 0.05% SeNP all displayed significant variations in MDA content over the SS control, with *p*-values of less than 0.001, less than 0.001, and 0.025, respectively. Similarly, the SS experimental group treated with 0.1% SeNP alone and 0.1% SeNP in combination with 0.05% SeNP exhibited significant variations in MDA content over the SS control, with *p*-values of less than 0.001 and less than 0.001, respectively. However, no significant variation in MDA content was noted between the SS control and the SS experimental group treated with 0.1% SeNP and 0.01% SeNP (*p* = 0.018) (Fig. 6B).

### Hydrogen peroxide

No significant variations in hydrogen peroxide (H_2_O_2_) content were noted between the NoSS control and the NoSS experimental groups treated with 0.05% SeNP, 0.01% SeNP, or the combination of 0.01% SeNP and 0.05% SeNP under the NoSS condition (*p* > 0.05). Similarly, no significant variations in H2O2 content were found between the SS control and the SS experimental groups treated with 0.05% SeNP, 0.01% SeNP, or the combination of 0.01% SeNP and 0.05% SeNP under the SS condition (*p* > 0.05). However, within the SS condition, significant variations in H2O2 content were noted. The SS experimental group treated with 0.01% SeNP displayed a significantly higher H2O2 content over the SS control, with *p*-value less than 0.01. Additionally, the SS experimental group treated with the combination of 0.01% SeNP and 0.05% SeNP showed a statistically significant variation in H2O2 content over the SS control, with *p*-value 0.022. Furthermore, the SS experimental group treated with 0.1% SeNP alone and in combination with 0.05% SeNP both exhibited significantly higher H2O2 content over the SS control, with *p*-values of less than 0.001 and less than 0.001, respectively. Additionally, the SS experimental group treated with the combination of 0.1% SeNP and 0.01% SeNP displayed significantly higher H_2_O_2_ content over the SS control, with *p*-value less than 0.01. These findings suggest that the application of SeNPs, particularly at specific concentrations under salinity stress conditions, can modulate the H2O2 content in plants. The results indicate that SeNPs may influence the production of reactive oxygen species and oxidative stress responses in plants, highlighting their potential role in plant defense mechanisms against salinity-induced oxidative damage (Fig. 6C).

**Fig. 7:**
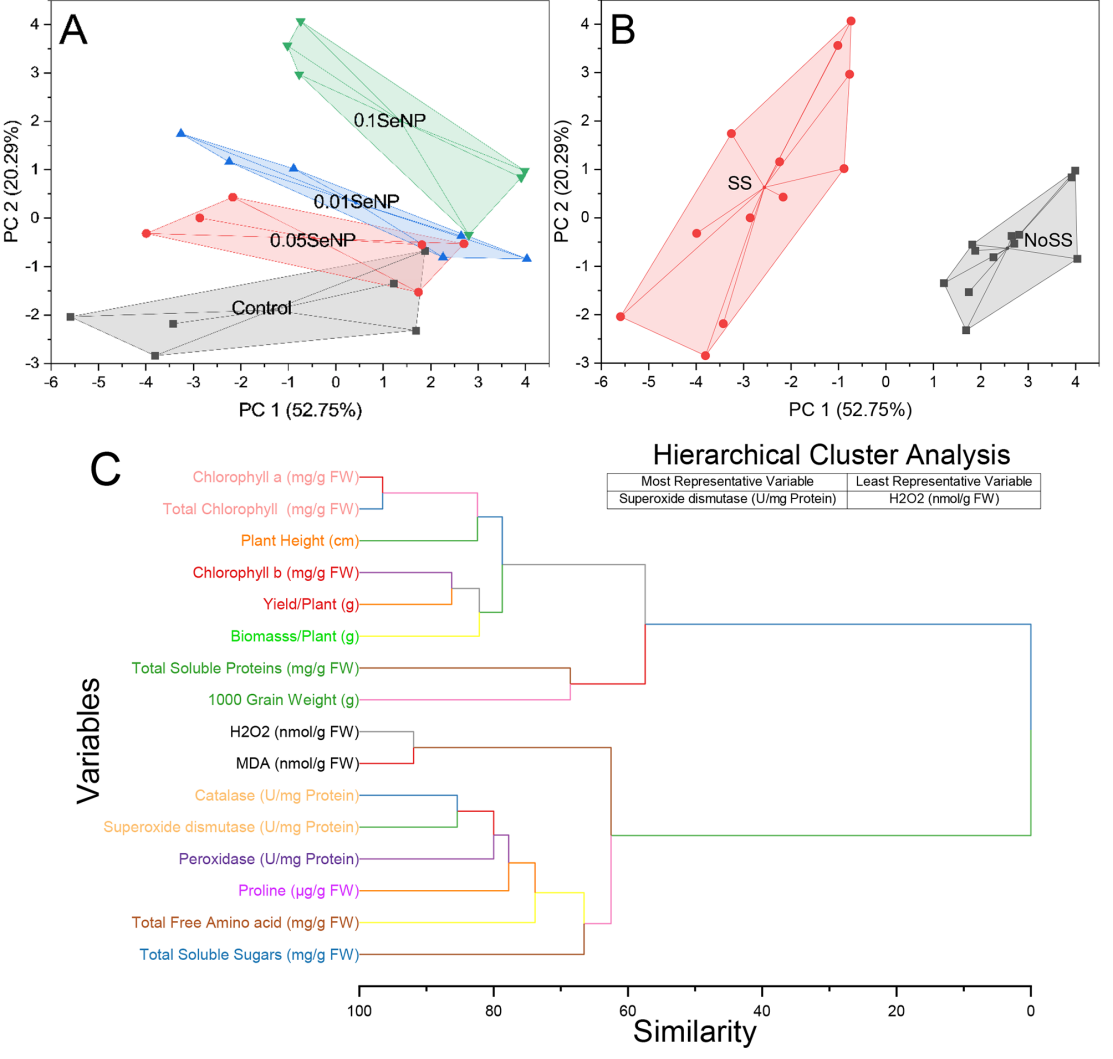
Cluster plot with convex hull for SeNP **(A)**, salinity stress **(B)** and hierarchical cluster plot for studied attributes **(C)**

The control cluster exhibits proximity among points characterized by coordinates such as (1.8836, -0.67912), (1.69031, -2.32132), (1.22389, -1.34951), and others. The 0.05% SeNP cluster encompasses points denoted by (1.7486, -1.53412), (1.82026, -0.55258), (2.70553, -0.53308), and more, indicating their association with the 0.05% SeNP category. In the 0.01% SeNP cluster, points such as (2.2673, -0.81394), (2.64986, -0.37447), (4.03619, -0.84702), and others manifest close proximity, signifying their classification as 0.01% SeNP. The 0.1% SeNP cluster comprises points including (2.81082, -0.35018), (3.9151, 0.83544), (3.99095, 0.97508), and more, which demonstrate adjacency, indicative of their membership in the 0.1% SeNP category (Fig. 7A). Cluster 1 (NoSS) consists of several points with coordinates such as (1.8836, -0.67912), (1.69031, -2.32132), (1.22389, -1.34951), and more. These points are scattered across the plot, and a convex hull is drawn around them, forming a polygon that encapsulates the cluster. Cluster 2 (SS) is represented by points like (-5.59553, -2.04089), (-3.80546, -2.84626), (-3.42874, -2.18605), and others. These points are located separately from Cluster 1, and a convex hull is drawn around them as well, creating a distinct polygon that encompasses the second cluster (Fig. 7B). The variables Chlorophyll a (mg/g FW) and Total Chlorophyll (mg/g FW) demonstrate a similarity coefficient of 3.45938, indicating a strong resemblance in their respective measurements. This suggests a close association between these variables, potentially reflecting their interconnected role in chlorophyll content assessment. Similarly, the variables H2O2 (nmol/g FW) and MDA (nmol/g FW) exhibit a similarity score of 8.07886. This moderate level of similarity implies a potential relationship between these variables, possibly indicating their involvement in oxidative stress responses or lipid peroxidation mechanisms. In contrast, the variables Chlorophyll b (mg/g FW) and Yield/Plant (g) demonstrate a similarity value of 13.75976. This suggests a somewhat weaker association between these variables, hinting at a potential influence of chlorophyll b content on the yield per plant. Furthermore, the variables Catalase (U/mg Protein) and Superoxide dismutase (U/mg Protein) showcase a similarity coefficient of 14.56703, implying a notable likeness in their measurements. This similarity underscores their potential correlation in antioxidant defense mechanisms, given their involvement in enzymatic activities related to reactive oxygen species. The variable Plant Height (cm) and an unidentified variable display a similarity score of 17.59582. This indicates a certain degree of resemblance, suggesting a potential relationship between plant height and the unidentified variable, which requires further investigation for identification. Likewise, the variables Biomass/Plant (g) and an unidentified variable exhibit a similarity value of 17.86124. This moderate level of similarity suggests a potential connection between these variables, perhaps pointing to their interdependence in assessing plant biomass per unit. Additionally, the variables Peroxidase (U/mg Protein) and an unidentified variable share a similarity score of 20.01115, indicating a noteworthy resemblance. This similarity hints at a potential association between peroxidase activity and the unidentified variable, necessitating further exploration. Moreover, the variables Proline (µg/g FW) and an unidentified variable demonstrate a similarity coefficient of 22.24545, indicating a substantial likeness. This suggests a potential relationship between proline content and the unidentified variable, underscoring the need for additional investigation to determine its nature. The variables Total Free Amino acid (mg/g FW) and an unidentified variable exhibit a similarity value of 26.15454, implying a significant resemblance. This points to a potential connection between the total free amino acid content and the unidentified variable, necessitating further research for identification and understanding. Furthermore, the variables Total Soluble Proteins (mg/g FW) and 1000 Grain Weight (g) share a similarity of 31.41099, indicating a notable association between these variables. This suggests a potential relationship between total soluble protein content and the weight of 1000 grains, potentially reflecting their interdependency in grain development and quality assessment. Finally, the variables Total Soluble Sugars (mg/g FW) and an unidentified variable exhibit a similarity value of 33.47375, signifying a substantial resemblance. This suggests a potential relationship between total soluble sugar content and the unidentified variable, warranting further investigation for identification and characterization (Fig. 7C).

## Discussion

The improvement of chlorophyll contents, total soluble protein, sugar, biomass, and yield in plants by selenium nanoparticles (SeNPs) in the context of decreasing salinity stress can be attributed to several mechanisms. Firstly, SeNPs possess antioxidant properties, which can counteract the harmful effects of salinity-induced oxidative stress [30]. Salinity stress leads to the accumulation of reactive oxygen species (ROS) in plant cells, causing damage to various cellular components, including chlorophyll molecules [31]. SeNPs can scavenge ROS and protect chlorophyll from oxidative damage, thereby preserving its content [32]. Secondly, SeNPs have been shown to enhance the activity of antioxidant enzymes in plants [33]. Salinity stress disrupts the balance between ROS production and antioxidant defense mechanisms, resulting in oxidative damage to chlorophyll molecules [34]. Application of SeNPs can upregulate the activity of enzymes such as catalase (CAT), superoxide dismutase (SOD), and peroxidase (POD), which play a vital role in detoxifying ROS. Superoxide dismutase converts superoxide radicals (O2-) into hydrogen peroxide and molecular oxygen. By diminishing the levels of highly reactive superoxide radicals, SOD helps to mitigate ROS-induced harm. Additionally, peroxidase enzymes effectively detoxify hydrogen peroxide by utilizing it as an oxidizing agent in various reactions. By utilizing the reducing power of substrates, peroxidases convert hydrogen peroxide into water, effectively neutralizing its detrimental effects [35]. Proline contributes to the stabilization of macromolecules and cellular structures. It can interact with proteins, nucleic acids, and membranes, preventing their denaturation or disruption under stressful conditions. Proline’s unique conformational properties and ability to form hydrogen bonds make it valuable in maintaining the structural integrity of biomolecules [36]. By enhancing the antioxidant enzyme activity, SeNPs help maintain the integrity of chlorophyll molecules and prevent their degradation. Furthermore, SeNPs have been reported to regulate ion homeostasis in plants under salinity stress [37]. On the other hand high salinity stress often interrupts the uptake and distribution of essential nutrients, including magnesium (Mg) and potassium (K), which are crucial for chlorophyll synthesis [38]. SeNPs can modulate ion transporters and channels, promoting the efficient uptake and translocation of essential nutrients. This ensures an adequate supply of Mg and K for chlorophyll biosynthesis, leading to increased chlorophyll content [38].

## Conclusion

It is concluded that use of 0.1% SeNPs application rate is a better approach for the alleviation of salinity stress compared to 0.05% and 0.01% SeNPs. Addition of 0.1% SeNPs can play an imperative role in enhancement of wheat growth attributes and chlorophyll contents under salinity stress. It can also regulate the antioxidants which can alleviate the stress induce by salinity in wheat. Growers are recommended to apply 0.1% SeNPs for achievement of maximum wheat production in salinity stress conditions. More investigations are suggested at field level on different cereal crops for declaration of 1Se NPs as one of the best amendments for mitigation of salinity stress. In conclusion, the application of 0.1% SeNPs at a higher rate proves to be a more effective approach in mitigating salinity stress compared to the application of 0.05% and 0.01% SeNPs. However, further investigations are needed at the field level, focusing on different cereal crops, to officially declare 0.1% SeNPs as one of the best amendments for mitigating salinity stress.

## Data Availability

All data generated or analysed during this study are included in this published article.
